# Cancer Incidence and Mortality among Firefighters: An Overview of Epidemiologic Systematic Reviews

**DOI:** 10.3390/ijerph18052519

**Published:** 2021-03-03

**Authors:** Elena Laroche, Sylvain L’Espérance

**Affiliations:** School of Administration Sciences, Université TELUQ, Quebec, QC G1K 9H6, Canada; syles@hotmail.com

**Keywords:** firefighters, contaminants, occupational health, carcinogens

## Abstract

Firefighters are exposed to carcinogens that may increase their risk of developing many types of occupational cancer. Many systematic reviews (SRs) have been produced with sometimes conflicting conclusions. In this overview of reviews, we aim to assess the conclusion consistency across the available systematic reviews on the cancer risk in firefighters. Literature searches were conducted in several indexed databases and grey literature to retrieve systematic reviews aiming to evaluate cancer incidence or cancer mortality in firefighters. Results from included SRs were analyzed according to the tumour site. Out of 1054 records identified by the search in the databases, a total of 11 SRs were ultimately included. The original studies (n = 104) analyzed in the SRs were published between 1959 and 2018. The results consistently reported a significant increase in the incidence of rectal, prostate, bladder and testicular cancers as well as mesothelioma and malignant melanoma in firefighters compared to the general population. The SRs also indicate that death rates from rectal cancer and non-Hodgkin’s lymphoma are higher among firefighters. Consistent SR results suggest that several types of cancer may be more frequent in firefighters than in the general population.

## 1. Introduction

Firefighters are exposed to many contaminants in the course of their daily duties. The results of some studies have associated exposure to hazardous substances with significant health problems such as cancers in firefighters. According to the International Agency for Research on Cancer (IARC) (2010), occupational exposure as a firefighter may be carcinogenic (Group 2B) [1]. Several original studies assessed the risk of cancer incidence or mortality in firefighters (see Appendix A). Firefighters are exposed to carcinogens in many ways: carcinogens arising from combustion, incidental to structural firefighting or arising from work as firefighter (for example, diesel exhaust) [2].

At fire sites, after fighting flames or changing their protective equipment, women and men are exposed to a variety of substances such as soot, polycyclic aromatic hydrocarbons (PAHs), volatile organic compounds (VOCs), acid gases and particulate matters. Since the publication in 1990 of the first review on cancer risk in firefighters [3], many systematic reviews (SRs) have been produced with sometimes conflicting conclusions. In this overview of reviews, we aim to assess the conclusion consistency across the available systematic reviews on the cancer risk in firefighters.

## 2. Materials and Methods

This review was performed according to Preferred Reporting Items for Systematic Reviews and Meta-Analyses (PRISMA) statement [4]. Details for this checklist are presented in Appendix B.

### 2.1. Search Strategy

The literature search was conducted using MEDLINE (PubMed), Embase, Cochrane Library, Centre for Reviews and Dissemination, Web of Science, CINAHL, PsycNet, ABI /INFORM Global and SCOPUS, from the inception of the database up to October 12, 2019, in order to identify systematic reviews or meta-analyses on the risk of cancer in firefighters. Evidence search was also conducted on grey literature to identify relevant publications. Bibliographies of included studies were reviewed to identify additional references of interest. The database search strategies and websites consulted in the grey literature search are presented in Appendix C.

### 2.2. Selection of the Systematic Reviews

As there are a multitude of reviews on cancer incidence or mortality related to firefighting as an occupation, only reviews presenting a systematic literature search methodology, published in English or French, were eligible. To be included, reviews also had to focus on cancer incidence in firefighter compared to general population and to present an aggregated risk of cancer or cancer mortality. The same eligibility criteria were applied whether evidence was identified through indexed databases or grey literature. Study identification and eligibility assessment was performed by two independent reviewers. All inconsistencies were resolved by discussion. A list of excluded studies and the reason for their exclusion is presented in Appendix D.

### 2.3. Assessment of Methodological Quality, Data Extraction and Analysis

The methodological quality and data extraction of the included studies were assessed by one researcher and validated by a second researcher. The methodological quality was assessed using the ROBIS tool [5]. A standardized form was developed in order to extract the following data: author’s name, country, years of literature search, inclusion and exclusion criteria, number of studies included, type of synthesis, performance of a meta-analysis, comparator, aggregated risk of cancer incidence or cancer mortality by cancer types. The primary outcomes are consistent in risk of cancer or cancer mortality in firefighters across the included systematic reviews. For each cancer type, a quantitative analysis of the risk was performed. No meta-analysis of the published risk was performed.

There was no formal evaluation of publication bias. However, methodological measures such as literature searches in several indexed databases, grey literature searches and the omission of search limits (i.e., study types or years of publication) were implemented to reduce the risk of publication bias. However, the authors are aware that limiting inclusion according to language may have increased the risk of publication bias. However, since the majority of the scientific literature is published in English, the authors felt that the publication bias caused by limiting selection to only English or French systematic reviews was minor. Interventionary studies involving animals or humans, and other studies that require ethical approval, must list the authority that provided approval and the corresponding ethical approval code.

### 2.4. Data Synthesis

Qualitative synthesis were performed. Due to the heterogeneity of the risk estimates across the reviews, data for each cancer type were not aggregated in a meta-analysis. Risk estimates from individual reviews were grouped and presented by biological system. Overall cancer incidence and cancer mortality rates were analyzed separately.

## 3. Results

**Figure 1:**
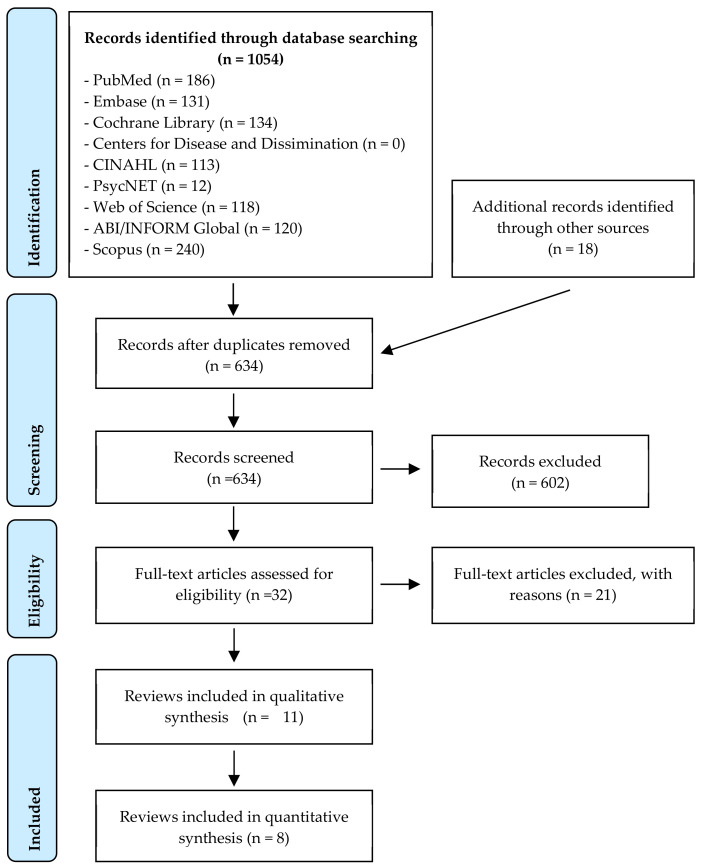
Evidence selection flow chart.

A total of 1072 abstracts were retrieved. After duplicate removal and abstract screening, 32 articles were eligible for full-text assessment, of which 11 systematic reviews were eligible for inclusion [1,2,3,6,7,8,9,10,11,12,13]. The study selection process is shown in Figure 1.

### 3.1. Description of Included Reviews

The main characteristics of the included systematic reviews are presented in Table 1. Briefly, the reviews were published by research teams from Canada [3,8,9,11,12], the United States [6,7], Australia [2], the United Kingdom [10], Iran [13] and Europe [1]. Six systematic reviews were published in a peer-reviewed journal [3,7,8,9,11,13] and five were published by a specific organization [1,2,6,10,11,12]. Years covered by the literature search varied among the included systematic reviews but generally covered from the inception of the indexed databases through 2018. One included review did not provide information about the years covered by the review [2]. Different inclusion and exclusion criteria were used in the included reviews. The reviews generally included epidemiological studies, observational studies or case-control studies reporting standardized cancer incidence rates or mortality rates among urban firefighters [1,2,3,6,7,8,9,10,11,12,13]. Four systematic reviews also considered published systematic reviews in their analysis [1,2,8,10]. Two reviews specified that they excluded studies on wildlife firefighters [12] and volunteer firefighters or trainees [13]. Specific inclusion and exclusion criteria were not reported in one review [1].

Overall, 104 different publications were included in the selected systematic reviews with a mean number of 28 (range: 2–48) per systematic review. The distribution of the different original studies is presented in Appendix A. These studies were published between 1959 and 2018 and consist in cohort studies (n = 47; 45.2%), case-control studies (n = 39; 37.5%), descriptive studies (n = 12; 11.5%), systematic reviews with or without meta-analysis (n = 5; 4.8%) and narrative reviews (n = 1; 1%) (Table 2). Most of the studies were published between 1990 and 2018 (n = 88; 84.6%). Global original study redundancy among included systematic reviews is estimated at 59.6%. Indeed, 62 original studies were included in at least two different systematic reviews. One study, Vena and Fiedler (1987) [15], was included in 10 systematic reviews.

A descriptive synthesis of the included studies was performed by three systematic reviews [2,9,12] while a quantitative synthesis of primary data was performed by seven reviews [3,6,7,8,10,11,13]. One systematic review performed both descriptive and quantitative synthesis [1]. Aggregated results of standardized cancer incidence rates or standardized cancer mortality rates for different types of cancer were reported in eight systematic reviews [1,3,6,7,8,10,11,13]. Types of cancer analyzed include lung, colon and rectum, brain and nervous systems, melanoma, multiple myeloma, testicular, prostate, stomach, skin, non-Hodgkin’s lymphoma, mouth and pharynx, esophagus, liver, pancreas, larynx, bladder, kidney, Hodgkin’s disease, leukemia, mesothelioma, breast (male), thyroid, bones, soft tissues, eye and intestine.

### 3.2. Methodological Quality of Included Reviews

**Figure 2:**
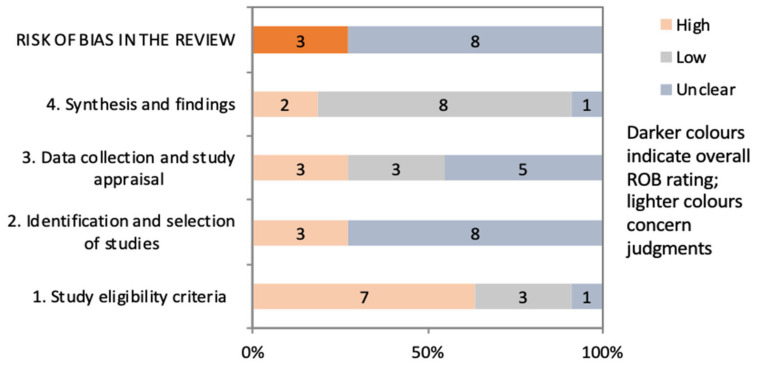
Concerns and risks of bias (using ROBIS tools) of the selected systematic reviews.

The methodological quality of the systematic reviews was assessed using the ROBIS tool (Figure 2). Overall, the risk of bias related to the literature search methodology was rated as high or uncertain in most of the identified reviews. Although most of the included reviews specify the inclusion and exclusion of original studies, few have a comprehensive literature search methodology for searching multiple indexed databases and grey literature. In addition, the methodology for selecting original studies is uncertain or poorly reported in the majority of systematic reviews. Few reported that the study identification, selection and data extraction was performed by two independent reviewers. The methodological quality of included studies was rarely assessed in the selected reviews. Only the publications by Sritharan et al. (2017) [11], IRSST (2018) [12] and Jalilian et al. [13] reported a methodological quality assessment of their included studies.

The original studies included in the selected review were generally well described and their results were aggregated using an appropriate statistical method. The methodological quality of these studies varied [11,12,13]. Heterogeneity was often reported across the studies and the presence of confounding biases was raised by different authors [1,2,7,8,11,12,13]. With the exception of the systematic reviews of Sritharan et al. [11] and Jalilian et al. [13], the possibility of publication bias was not evaluated.

### 3.3. Overall Risk of Cancer and Mortality by Cancer in Firefighters

Five SRs reported overall cancer risk or cancer mortality risk estimates among firefighters (Table 3) [3,7,8,9,14]. Global cancer incidence rates were reported in three SRs [8,9,14]. The results of the systematic reviews suggest that cancer incidence rates among firefighters do not differ significantly from that of a reference population (SIR varying from 0.89 to 1.02). Concerning the overall cancer mortality risk estimates, included SRs reported aggregated estimates ranging from 0.92 to 1.09 [3,6,7,8,13]. The results of three SRs indicated a significant increase from 4% to 9% of the standardized mortality rate in firefighters compared to a reference population [6,7,8].

### 3.4. Summary of Existing Estimates of Cancer Incidence and Mortality by Cancer in Firefighters according to the Tumour Site

#### 3.4.1. Respiratory Tract Cancers

Eight SRs analyzed the risk of cancer or cancer mortality related to specific tumour sites in the respiratory tract [1,2,3,6,7,10,12,13]. In its review, the IARC reviewed 11 individual cohort studies and three individual case-control studies on the risk of respiratory cancers in firefighters [1]. Few of the studies reported significant increases of cancer incidence or cancer mortality in this population. The Guidotti review found that evidence was sufficient to recognize an elevated cancer risk of lung cancer in firefighters [2]. However, Guidotti stated that this risk increase is likely to be heavily obscured by confounding factors such as smoking history and may not be as strong as would be suggested by the toxicological literature. Concerning the risk of mesothelioma, Guidotti concluded that the weight of the available evidence strongly favors the conclusion that mesothelioma is an occupational disease of firefighters [2]. An elevated risk of nasal sinus cancer in firefighters was also suggested in this SR, though they found that there was not sufficient evidence to make a conclusion on the risk of laryngeal cancer in firefighters [2]. In the SR published by the IRSST, non-convergent results were observed in the study analyzed concerning risk of head and neck cancer (including larynx, pharynx and nasopharynx cavities) and lung cancer in the firefighter population [12]. The authors concluded that the statistical association between firefighting and the risk of cancer is low for head and neck cancer and low to moderate for lung cancer. However, the IRSST review recognized that the available evidence on the risk of mesothelioma in firefighter presents convergent results that lead the authors to conclude that there may be a strong association between firefighting and incidence of mesothelioma in this population. Five SRs published aggregated results on the respiratory tract cancer incidence or cancer mortality in firefighters (Table 4) [3,6,7,10,13]. Published estimates in these reviews suggested that no significant increase of lung cancer, mouth and pharyngeal cancer or laryngeal cancer incidence or mortality is observed in firefighters compared to a reference population. Results from the Jalilian et al. SR, however, suggested an significant increase of mesothelioma (SIR = 1.60 (1.09–2.34)) in firefighters compared to the general population [13].

#### 3.4.2. Digestive Cancers

Eight SRs reported results on the risk of digestive cancers in firefighters [2,3,6,7,8,10,12,13]. Six of these reviews aggregated the results from original studies [3,6,7,8,10,13], while two presented a descriptive analysis [2,12].

In his SR, Guidotti reported that the literature generally supports the conclusion that there is an increased risk of colon cancer in firefighters [2]. He indicated that an assessment of causal factors in firefighters should be conducted in order to make a recommendation on cancer risk in this specific population. Although it is reported that rectal cancer would generally have the same risk factors as colon cancer, Guidotti made no conclusion as to the possibility of a link between being a firefighter and the development of rectal cancer in his review. The author also reported that the results of some original studies suggest increased risk for other types of cancer such as cancer of the esophagus, stomach, pancreas, small intestine, liver or biliary tract but so far without confirmation or replication in the firefighter population. Guidotti presented no conclusion for these cancer types.

In its SR, the IRSST reported various conclusions concerning the risk of digestive cancers in firefighters [12]. Based on the observation of results that are inconsistent from one study to another, the IRSST concluded that the degree of statistical association between the firefighting profession and colorectal or oesophageal cancer is mixed. The statistical association observed for gastric and pancreatic cancer was limited and nil, respectively.

Six SRs published aggregated results on the digestive cancer incidence or cancer mortality in firefighters (Table 5). Significant increases of cancer incidence was reported by two SRs for colon cancer [10,13], one SR for gastric cancer [7] and two SRs for rectal cancer [10,13]. Concerning cancer mortality, elevated rates were reported by two SRs for colon cancer [6,7], one SR for gastric cancer [6], three SRs for rectal cancer [6,7,13] and one SR for pancreatic cancer [6].

#### 3.4.3. Skin Cancers

Seven SRs reported conclusions on the skin cancer risk in firefighters [2,3,6,7,10,12,13]. In Guidotti’s SR, the author reported that firefighters are exposed to carcinogenic chemicals present in fire smoke, such as polycyclic aromatic hydrocarbons (PAH), that are known to be associated with skin cancer [2]. By reviewing the results from different original studies, the author suggested that melanoma incidence may be elevated in firefighters. He also reported that the available evidence of elevated risk of other types of skin cancer (i.e., basal and squamous cell carcinoma) is not sufficient to make a provisional recommendation on the general causation of these types of cancers with respect to firefighting [2]. In the IRSST review, the authors reviewed six original studies that reported data principally on melanoma incidence rate [12]. They judged that the degree of statistical association between the firefighting profession and melanoma is mixed and limited for other types of skin cancer. There was a weak to moderate level of evidence associated with these conclusions [12].

Five SRs reported pooled estimates on melanoma and other types of skin cancers (Table 6) [3,6,7,10,13]. Two SRs found an significant increase of melanoma incidence [10,13] and melanoma mortality [3,7] in firefighters. For other types of skin cancer, the SR by Crawford et al. reported a significant increase of skin cancer incidence, at 30% [10], while the SR by Jalilian et al. found no significant results [13]. As for skin cancer mortality, two SRs reported an elevated risk of mortality in firefighters [6,7] and one did not [13].

#### 3.4.4. Haematological Cancers

Nine SRs reported results about haematological cancer incidence or mortality rates [1,2,3,6,7,8,10,12,13]. Guidotti reviewed several original studies on the risk of non-Hodgkin’s lymphoma, leukemia and myeloma in firefighters [2]. He found that the evidence is sufficient to conclude a positive association between firefighting and incidence of lymphoma and leukemia [2]. Elevated risk of myeloma in firefighters was suggested and required more validation before conclusions could be made on general causation for this cancer. The IRSST review analyzed studies on the risk of multiple myeloma, non-Hodgkin’s lymphoma and leukemia [12]. They judged that the degree of statistical association between the firefighting profession and haematological cancers varies from limited to mixed [12]. The level of evidence associated with these conclusions is very weak to moderate [12].

Seven SRs reported pooled results of the incidence or mortality of haematological cancers in firefighters (Table 7) [1,3,6,7,8,10,13]. Compared to a standardized population, significant increases in non-Hodgkin’s lymphoma mortality in firefighters were consistently reported in the included SR [1,6,7,8,10,13]. No significant difference was generally reported for multiple myeloma, Hodgkin’s lymphoma and leukemia among firefighters [1,3,6,7,8,10,13].

#### 3.4.5. Urogenital Cancers

Nine SRs published results about the risk of urogenital cancers or mortality by genitourinary cancers in firefighters [1,2,6,7,8,10,11,12,13]. In Guidotti’s SR, conclusions were based on the analysis of several original studies as well as reviews [2]. It was concluded that the available evidence is sufficient for making recommendation of an elevated risk of bladder cancer, kidney cancer and testicular cancer in firefighters [2]. However, the evidence reviewed by Guidotti did not allow him to conclude that there was an elevated risk of prostate cancer in this population [2]. Different results were published in the IRSST SR [12], the authors of which judged that the degree of statistical association between the firefighting profession and prostate cancer, kidney cancer and bladder cancer is mixed or limited [12]. The level of evidence associated with these conclusions is weak to moderate [12]. They also found no statistical association between firefighting and testicular cancer [12].

Seven SRs reported pooled estimates of the risk of urogenital cancers in firefighters (Table 8). A significant increase of the incidence of prostate cancer [7,10,11,13], bladder cancer [8,10,13] and testicular cancer [1,7,13] was consistently reported in the SRs. Inconsistent risk estimates were reported among included SRs for all urogenital cancer mortality rates and kidney cancer incidence rates.

#### 3.4.6. Other Types of Cancers

Eight SRs studied the risk of brain and nervous system cancer [1,3,6,8,9,10,12,13], male breast cancer [1,12,13], thyroid cancer [1,12,13], bone cancer [1,12,13], soft tissue sarcoma [13] and eye cancer [13]. Guidotti’s SR reviewed evidence on the risk of brain cancer, male breast cancer and thyroid cancer in firefighters [2]. He concluded that the weight of evidence suggests that firefighting may be associated with elevated risk for brain cancer, especially gliomas, in certain subgroups of patients. However, he mentioned that the risk estimates for this cancer are diluted by inclusion of other tumour types that are not known to be associated with this occupation. Guidotti did not come to a firm conclusion about male breast cancer risk in firefighters. However, he did report that there are four plausible explanations that may explain the occurrence of male breast cancer in firefighters: (1) exposure to potent carcinogens produced by product combustion; (2) stimulation of male breast tissues by estrogen-like chemicals such as numerous polycyclic aromatic hydrocarbons and chlorinated polycyclic hydrocarbons; (3) shiftwork; and (4) electromagnetic fields created by radio transmission and electronic equipment used by firefighters. Finally, based on the analysis of several original studies, Guidotti concluded that there is insufficient evidence to link thyroid cancer to firefighting. The IRSST reported conclusions about the risk of brain cancer, male breast cancer, thyroid cancer and bone cancer [12]. With respect to brain cancer, the IRSST reviewed seven original studies that all individually reported results on cancer incidence in firefighters. Half of them reported significant increases of brain cancer in firefighters. Based on the results, the IRSST concluded that the degree of statistical association is mixed and associated with a low level of evidence [12]. For male breast cancer and thyroid cancer, the IRSST found no data that indicated an association between the incidence of these cancers and the firefighting profession [12]. Finally, Gomes et al. examined the role of different occupational and environmental risk factors such as firefighting in the risk of brain cancer [9]. Based on two studies, they found that firefighting as an occupation may be associated with increased risk of brain cancer.

Five SRs aggregated results from original studies [3,6,8,10,13]. Globally, inconsistent risk estimates were reported among the different SRs for brain and nervous system cancer (Table 9). Based on 10 studies, Jalilian et al. found a significant increase of thyroid cancer in firefighters [13].

## 4. Discussion

The main objective of this overview was to assess the consistency of the conclusions made in the available systematic reviews on the cancer risk in firefighters. Analysis of the cancer rates published in the different SRs indicated that incidence rates of rectal cancer, prostate cancer, bladder cancer, testicular cancer, mesothelioma and malignant melanoma are consistently reported as higher in firefighters compared to the general population. The results of the SRs also indicated that death rates from rectal cancer and non-Hodgkin’s lymphoma are higher among firefighters.

However, these observations must be interpreted with caution. This literature has many limitations and shortcomings. One limitation is that the methodological quality of the reviewed SRs is generally low. For the most part, the inclusion and exclusion criteria for original studies are absent or not very explicit. The literature search methodology is generally poorly described. For the majority of the SRs, there is minimal information given regarding the number of databases in which studies were searched, the keywords used to conduct the literature search, and the method for selecting, assessing and extracting study data. In several SRs, it appears that only one reviewer handled the identification of relevant studies, the final study selection and the data extraction [3,6,8,9,10] Very few SRs also searched for relevant literature in the grey or unpublished literature [2,3]. All of these concerns raise the possibility that there may be a selection bias in the SRs identified. Moreover, except for Jalilian [13], none of the reviews included a formal assessment of the presence or absence of publication bias.

Of the SRs analyzed, only two performed an analysis of the methodological quality of the included studies using a validated measurement tool [12,13]. In the IRSST SR, the authors analyzed the methodological quality of the studies by using the Newcastle-Ottawa scale [12]. However, the authors of the IRSST report did not use the results of these analyses to formulate conclusions because of the various limitations observed in the use of this assessment tool for occupational epidemiologic studies [12]. Jalilian et al. also used the Newcastle-Ottawa scale to analyze the methodological quality of the studies identified [13]. The authors considered the majority of the studies they included in their review to be of good methodological quality. The impact of this result also does not, however, appear to have been discussed and considered by the authors of this review. The extent to which a systematic review can draw conclusions about the effects of an intervention depends on the validity of the data and results from the included studies. In particular, a meta-analysis of invalid or low-quality studies may produce a misleading result, yielding a narrow confidence interval around the wrong intervention effect estimate [16]. Variations in study quality can explain differences in the findings of studies that are included in a systematic review. As a result, the quality of a study will affect the strength of the evidence that can be drawn from it. In other words, it determines whether we can be confident that the results of a study reflect the ‘truth’ and by extrapolation, whether we can be confident in the results of the systematic review [16,17].

Of the included SRs, eight aggregated the results of the original studies [1,3,6,7,8,10,11,13]. Considering the presence of heterogeneity, both at the contextual level and statistical level, the decision to combine the results of the original studies in a meta-analysis is methodologically questionable. Indeed, differences in the populations included, the degree of exposure to the various carcinogens present during firefighting, the effect measure chosen (SMR, SIR, PMR, RR, OR) and the reference populations make it difficult to compare the results of the original studies. Several confounding factors may also have an effect on the cancer incidence rates or cancer mortality rates. Factors such as the origin of the subjects, the number of years of service as a firefighter and the worker’s basal health may have influenced the results. Since many of the original studies are based on mortality registers that sometimes span several decades, improvements in fire control practices and changes in the worker’s personal protective equipment could potentially have had an impact on the observed results. Exposure to the various carcinogens present during a fire may also have changed over time. For example, in the past, many buildings contained asbestos, and firefighters who fought fires in this type of building were more often exposed to this carcinogen and may have been at higher risk of mesothelioma than they are today, when this material is increasingly banned in new buildings. Finally, considering the differences between the original studies and the possible confounding factors, a more descriptive analysis of the current evidence, such as that carried out by Guidotti [2], IRSST [12] or IARC [1] seems to be more appropriate for this research topic.

The results of our review show that, statistically, some cancers may be more prevalent or may cause more deaths in male firefighters. In order to determine a causal relationship, it is important that a biological mechanism or exposure to a recognized carcinogen be linked to the observed cancers. Systematic reviews by Guidotti and the IRSST presented several explanations of the biological plausibility of the occurrence of cancers in firefighters [2,12]. Although the links between exposure to different carcinogens such as asbestos, organic solvents or PAHs and the incidence of bladder cancer, mesothelioma or non-Hodgkin’s lymphoma have been more thoroughly studied, the biological plausibility for other cancers needs to be better investigated. For example, with respect to the risk of rectal cancer in firefighters, the available epidemiological data do not provide sufficient evidence on the etiological role of firefighter employment in the incidence of or mortality from colon or rectal cancer [18]. For prostate cancer, there are some plausible hypotheses regarding gene-environment interactions in hormone synthesis, action and metabolism, although no specific environmental pollutants have been identified [12]. But, in his SR, Guidotti mentions that prostate cancer, despite a number of studies that appear to suggest an excess, is an example of a diagnosis that does not fit the logical framework required for a presumption [2]. On the face of it, the evidence would seem to suggest a rather weak association with toxicological plausibility [2]. He also mentions that the detailed examination of the problem, however, suggests that this is a spurious association caused by screening bias, which is exceptionally strong in this case [2]. It is important to keep in mind that prostate cancer is one of the most frequently diagnosed cancers in men. According to the Canadian Cancer Society, about one in nine men will be diagnosed with prostate cancer during his lifetime [19]. Canadian cancer statistics also report that one in 29 male Canadians will die from it [19]. Finally, concerning the risk of testicular cancer in firefighters, no plausible biological mechanism has so far been proposed for the association between testicular cancer and firefighting, and no specific agent or agents identified as risk-conferring [20].

As with every review, our work has its strengths and limitations. To the best of our knowledge, it is the first overview of the SRs on the risk of cancer in firefighters. To carry out this project, we used recognized literature search methods in order to minimize the risk of bias in our methodology. Most of the results deal with to the risk of cancer in male firefighters. However, more and more women are working as firefighters and there is currently little evidence available regarding the risk of cancer in female firefighters. A study published in 2020 on cancer risks among career male and female Florida firefighters indicates that female firefighters showed significantly elevated risk of brain and thyroid cancers and an elevated risk of melanoma that approached statistical significance [21]. More studies are needed to understand the occupational cancer risk in this population.

From a methodological point of view, we observed that the methodological quality of the included systematic reviews varies from low to moderate. While it is true that some steps in conducting a systematic review are missing in the included reviews, the final results of the methodological quality assessment may be affected by the methodological quality analysis tool used (ROBIS). These tool do not seem well suited to adequately assessing the methodological quality of systematic reviews of epidemiological studies. Those analytical tools are more suitable for randomized control trials (RCT) or non-randomized study of intervention (NRSI). An adaptation of the PRISMA guidelines for meta-epidemiological studies have been published in 2017 [22] but did not provided criteria for assessing methodological quality of this type of study.

## 5. Conclusions

An analysis of existing systematic reviews concerning the risk of cancer or cancer mortality in firefighters found that the incidence of rectal cancer, prostate cancer, bladder cancer, testicular cancer, mesothelioma and malignant melanoma are consistently reported as significantly higher in this population compared to the general population. The results of SRs also indicate that death rates from rectal cancer and non-Hodgkin’s lymphoma are higher among firefighters. However, caution should be exercised in interpreting these results due to the low methodological quality of the SRs reviewed. It is also not possible to generalize these results to all firefighters since the original studies focused on cancer risk among male firefighters. More studies are needed to determine the cancer risk among female firefighters.

## Figures and Tables

**Table 1:** Main characteristics of the included systematic reviews.

Authors (Year) [Ref] Country	Literature Search	Inclusion Criteria	Exclusion Criteria	N Studies	Type of Synthesis	Meta-Analysis Performed
Howe and Burch (1990) [3] Canada	NR	Epidemiologic studies on the risk of cancer in firefighters.	Epidemiologic studies relevant to the topic containing major bias.	12	Quantitative	Yes
Samet and Bhavsar (2005) [6] United States	From January 1966 through September 2004	Studies reporting cancer risk estimates for urban firefighters; Studies that addressed risks of harmful exposures encountered while performing firefighting duties; Studies published in English.	Studies on wildland firefigthers; Studies not written in English; Duplicate reports on the same study group.	18	Quantitative	Yes
Youakim S. (2006) [8] Canada	From 1966 through 2005	Studies reporting results on incidence of brain cancer, colon cancer, bladder cancer, leukemia and non-Hodgkin’s lymphoma in firefighters.	NR	26	Quantitative	Yes
LeMaster et al. (2006) [7] United-States	From inception through December 2003	Standardized mortality ratio (SMR), proportional mortality ratio (PMR), relative risk (RR), standardized incidence ratio (SIR), and case-control/mortality odds ratio (OR) studies related to firefighters and cancer risk, published in English; Firefighters included in studies must have at least one year of service except for studies basing employment on death certificates.	Abstracts and reviews.	28	Quantitative	Yes
IARC (2010) [1] Europe	NR	NR	NR	46	Descriptive Quantitative	Yes (only for 3 cancer types)
Gomes et al. (2011) [9] Canada	From inception through the end of 2010	Studies in English on adult brain cancer etiology in males and females or that identified and reported risk of brain neoplasm from exposure to occupational and/or environmental factors.	Randomized or non-randomized clinical studies; Clinical studies that reported on diagnostic, therapeutic or outcome; Editorials or commentaries on disease states; Research articles on animal experiments; Studies not written in English; Studies on children (under 19 of age).	2	Descriptive	No
Guidotti, T. (2014) [2] Australia	NR	NR	NR	43	Descriptive	No
Crawford et al. (2017) [10] UK	Primary studies of [14] + new studies from 2009	Studies containing usable data of risk of cancer in firefighters, published in English.	NR	41	Quantitative	Yes
Sritharan et al. (2017) [11] Canada	From January 1980 through December 2017	Studies that reported results for original case-control or cohort studies that contained specific job titles related to ever/never firefighting and police work; Studies that examined associated prostate cancer incidence and/or mortality using any type of relative risk estimator: hazard ratio (HR), odds ratio (OR), relative risk (RR), standardized mortality ratio (SMR), or standardized incidence ratio (SIR) with corresponding 95% confidence intervals.	Reviews, meta-analyses, editorials and experimental studies that reported risk estimates that were based only on internal comparisons between different occupational groups rather than on comparisons to the general population.	26	Quantitative	Yes
IRSST (2018) [12] Canada	From 2017	Studies reporting results on cancer risk in firefighters, published in English or French and peer reviewed.	Case reports and studies on wildlife firefighters.	25	Descriptive	No
Jalilian et al. (2019) [13] Iran	From inception through January 1, 2018	Original articles published in English in peer-reviewed journals that investigated the association of firefighting with any type of cancer risk or cancer mortality in humans; Studies reporting a relative risk (RR), odds ratio (OR), standardized incidence ratio (SIR) or standardized mortality ratio (SMR) with 95% confidence intervals (CI) or providing sufficient data to calculate them; Studies where the exposure clearly preceded the outcome and the firefighting occupation was compared to the general population and other occupations or an internal comparison was conducted.	Studies on volunteer or trainee firefighters, and those reporting effect size on cancers in organ systems of the body only.	48	Quantitative	Yes

**IARC:** International Agency for Research on Cancer; **IRSST:** Institut de recherche Robert-Sauvé en santé et en sécurité du travail; **NR:** non-reported.

**Table 2:** Principal characteristics of the primary studies included in the selected systematic review (n = 104).

Characteristics	N (%) (Total = 104)
**Date of publication**	
1951–1960	1 (1)
1961–1970	0 (0)
1971–1980	5 (4.8)
1981–1990	15 (14.4)
1991–2000	26 (25.0)
2001–2010	29 (27.9)
2011–2020	28 (26.9)
**Study types**	
Systematic review/meta-analysis	5 (4.8)
Cohort	47 (45.2)
Case-control	39 (37.5)
Descriptive	12 (11.5)
Other	1 (1)
**Citation frequencies in SR**	
1	42 (40.4)
2–4	31 (29.8)
5–9	30 (28.8)
10–12	1 (1.0)
Redundancy rate	62 (59.6)

SR: systematic review.

**Table 3:** Summary of the overall cancer incidence rates and overall cancer mortality rates in included systematic reviews.

Authors (Year) [Ref]	Cancer Incidence		Cancer Mortality	
	N Study	Risk Estimates (95% CI)	N Study	Risk Estimates (95% CI)
Howe and Burch (1990) [3]	----	----	5	SMR = 0.92 (0.79–1.07)
Samet and Bhavsar (2005) [6]	----	----	2	**SMR = 1.09 (1.06–1.11)**
LeMaster et al. (2006) [7]	----	----	13	**SMR = 1.05 (1.00–1.09)**
Youakim S. (2006) [8]	4	sRR = 1.02 (0.93–1.11)	15	**sRR = 1.04 (1.02–1.07)**
Jalilian et al. (2019) [13]	12	SIRE = 0.89 (0.93–1.05)	22	SMRE = 0.99 (0.92–1.06)

**SIRE ^1^:** Summary incidence ratio estimate; **SMR:** standardized mortality ratio; **SMRE ^1^**: summary of mortality ratio estimate; **sRR:** summary relative risk. **Significant results are in bold.** ^1^: For Jalilan et al. (2019) [13], «summary incidence risk estimates (SIREs) and summary mortality risk estimates (SMREs) were calculated for each cancer outcome by pooling risk estimates (SIRs, ORs and RRs) and mortality estimates (SMRs, ORs, RRs), respectively»).

**Table 4:** Summary of risk estimates of respiratory tract cancers in firefighters from included SR.

Authors (Year) [Ref]	Cancer Incidence			Cancer Mortality	
	N Study	Risk Estimates (95% CI)	N Study		Risk Estimates (95% CI)
**Lung cancer**					
Howe and Burch (1990) [3]	----	----	6		SMR = 0.92 (0.79–1.07)
LeMaster et al. (2006) [7]	----	----	19		sRR = 1.03 (0.97–1.08)
Crawford et al. (2017) [10]	21	RR = 0.91 (0.82–1.01)	----		----
Jalilian et al. (2019) [13]	17	SIR = 0.94 (0.84–1.06)	17		SMR = 1.00 (0.92–1.09)
**Mouth and pharyngeal cancer**					
Samet and Bhavsar (2005) [6]	----	----	NR		SMR = 1.22 (0.92–1.61)
LeMaster et al. (2006) [7]	----	----	5		sRE= 1.23 (0.96–1.55)
Jalilian et al. (2019) [13]	11	SIR = 1.15 (0.91–1.44)	11		SMR = 1.21 (0.95–1.55)
**Laryngeal cancer**					
Samet and Bhavsar (2005) [6]	----	----	NR		SMR = 1.11 (0.80–1.55)
LeMaster et al. (2006) [7]	----	----	3		sRE= 1.22 (0.87–1.70)
Crawford et al. (2017) [10]	8	RR = 1.05 (0.67–1.65)	----		----
Jalilian et al. (2019) [13]	10	SIR = 0.93 (0.66–1.30)	4		SMR = 0.74 (0.48–1.15)
**Mesothelioma**					
Jalilian et al. (2019) [13]	5	**SIRE = 1.60 (1.09–2.34)**	----		----

**RR:** relative risk; **SIR:** standardized incidence ratio; **SIRE:** summary incidence ratio estimate; **SMR:** standardized mortality ratio; **sRE:** summary risk estimate; **sRR:** summary relative risk. **Significant results are in bold.**

**Table 5:** Summary of risk estimates of digestive cancers in firefighters from included SR.

Authors (Year) [Ref]	Cancer Incidence		Cancer Mortality	
	N Study	Risk Estimates (95%CI)	N Study	Risk Estimates (95%CI)
**Colon cancer**				
Howe and Burch (1990) [3]	----	----	3	SMR = 1.12 (0.77–1.57)
Samet and Bhavsar (2005) [6]	----	----	NR	**SMR = 1.16 (1.06–1.26)**
LeMaster et al. (2006) [7]	----	----	25	**sRR = 1.21 (1.03–1.41)**
Youakim S. (2006) [8]	5	sRR = 1.06 (0.84–1.32)	10	sRR = 1.07 (0.95–1.18)
Crawford et al. (2017) [10]	13	**mRR = 1.18 (1.08–1.29)**	----	----
Jalilian et al. (2019) [13]	10	**SIRE = 1.14 (1.06–1.23)**	13	SMRE = 1.10 (0.91–1.34)
**Gastric cancer**				
Samet and Bhavsar (2005) [6]	----	----	NR	**SMR = 1.22 (0.92–1.16)**
LeMaster et al. (2006) [7]	**13**	**sRR = 1.22 (1.04–1.44)**	----	----
Crawford et al. (2017) [10]	16	mRR = 0.93 (0.78-1.12)	----	----
Jalilian et al. (2019) [13]	13	SIRE = 1.04 (0.90–1.20)	16	SMRE = 1.03 (0.92–1.15)
**Rectal cancer**				
Samet and Bhavsar (2005) [6]	----	----	NR	**SMR = 1.36 (1.17–1.57)**
LeMaster et al. (2006) [7]	----	----	13	**sRR = 1.29 (1.10–1.51)**
Crawford et al. (2017) [10]	12	**mRR = 1.16 (1.05–1.29)**	----	----
Jalilian et al. (2019) [13]	10	**SIRE = 1.09 (1.00–1.20)**	12	**SMRE = 1.36 (1.18–1.57)**
**Esophageal cancer**				
Samet and Bhavsar (2005) [6]	----	----	NR	SMR = 1.01 (0.80–1.28)
LeMaster et al. (2006) [7]	----	----	8	sRR = 1.16 (0.86–1.57)
Crawford et al. (2017) [10]	13	mRR = 1.09 (0.83–1.42)	----	----
Jalilian et al. (2019) [13]	12	SIRE = 1.09 (0.87–1.37)	9	SMRE = 1.01 (0.76–1.34)
**Liver cancer**				
Samet and Bhavsar (2005) [6]	----	----	NR	SMR = 1.21 (0.95–1.54)
LeMaster et al. (2006) [7]	----	----	7	sRR = 1.04 (0.72–1.49)
Jalilian et al. (2019) [13]	9	SIRE = 0.93 (0.80–1.08)	11	SMRE = 1.05 (0.79–1.39)
**Pancreatic cancer**				
Samet and Bhavsar (2005) [6]	----	----	NR	**SMR = 1.16 (1.00–1.34)**
LeMaster et al. (2006) [7]	----	----	13	sRR = 1.10 (0.91–1.34)
Crawford et al. (2017) [10]	18	mRR = 1.03 (0.92–1.15)	----	----
Jalilian et al. (2019) [13]	14	SIRE = 1.09 (0.96–1.24)	11	SMRE = 1.13 (0.99–1.29)
**Intestine cancer**				
Jalilian et al. (2019) [13]	3	SIRE = 1.27 (0.89–1.82)	----	----

**mRR:** meta relative risk; **SIRE:** summary incidence ratio estimate; **SMR:** standardized mortality ratio; **SMRE**: summary of mortality ratio estimate; **sRR:** summary relative risk. **Significant results are in bold.**

**Table 6:** Summary of risk estimates of skin cancer in firefighters from included SR.

Authors (Year) [Ref]	Cancer Incidence		Cancer Mortality	
	N Study	Risk Estimates (95%CI)	N Study	Risk Estimates (95%CI)
**Malignant melanoma**				
Howe and Burch (1990) [3]	----	----	5	**SMR = 1.73 (1.00–2.74)**
LeMaster et al. (2006) [7]	----	----	10	**sRR = 1.32 (1.10–1.57)**
Crawford et al. (2017) [10]	10	**mRR = 1.41 (1.21–1.65)**	----	----
Jalilian et al. (2019) [13]	11	**SIRE = 1.21 (1.02–1.45)**	3	SMRE = 1.33 (0.98–1.81)
**Skin cancers**				
Samet and Bhavsar (2005) [6]	----	----	NR	**SMR = 1.49 (1.25–1.77)**
LeMaster et al. (2006) [7]	----	----	8	**sRR = 1.39 (1.10–1.73)**
Crawford et al. (2017) [10]	4	**mRR = 1.30 (1.08–1.57)**	----	----
Jalilian et al. (2019) [13]	4	SIRE = 1.12 (0.95–1.31)	4	SMRE = 1.08 (0.79–1.47)

**mRR:** meta relative risk; **SIRE:** summary incidence ratio estimate; **SMR:** standardized mortality ratio; **SMRE**: summary of mortality ratio estimate; **sRR:** summary relative risk. **Significant results are in bold.**

**Table 7:** Summary of risk estimates of haematological cancers in firefighters from included SR.

Authors (Year) [Ref]	Cancer Incidence		Cancer Mortality	
	N Study	Risk Estimates (95% CI)	N Study	Risk Estimates (95% CI)
**Multiple myeloma**				
Howe and Burch (1990) [3]	----	----	5	SMR = 1.51 (0.91–2.35)
LeMaster et al. (2006) [7]	----	----	10	**sRR = 1.53 (1.21–1.89)**
Crawford et al. (2017) [10]	10	mRR = 1.12 (0.99–1.27)	----	----
Jalilian et al. (2019) [13]	8	SIRE = 1.00 (0.83–1.23)	4	SMRE = 1.07 (0.83–1.37)
**Non Hodgkin lymphoma**				
Samet and Bhavsar (2005) [6]	----	----	NR	**SMR = 1.36 (1.18–1.58)**
LeMaster et al. (2006) [7]	----	----	8	**sRR = 1.51 (1.31–1.73)**
Youakim S. (2006) [8]	3	sRR = 1.34 (0.86–1.97)	8	**sRR = 1.40 (1.20–1.60)**
IARC (2010) [1]	----	----	7	**RE = 1.21 (1.08–136)**
Crawford et al. (2017) [10]	16	**mRR = 1.14 (1.05–1.23)**	----	----
Jalilian et al. (2019) [13]	14	SIRE = 1.07 (0.96–1.20)	8	**SMRE = 1.42 (1.05–1.90)**
**Hodgkin lymphoma**				
LeMaster et al. (2006) [7]	----	----	3	sRR = 1.07 (0.59–1.92)
Crawford et al. (2017) [10]	5	mRR = 1.18 (0.93–1.43)	----	----
Jalilian et al. (2019) [13]	8	SIRE = 1.12 (0.86–1.47)	4	SMRE = 1.21 (0.46–3.18)
**Leukemia**				
Samet and Bhavsar (2005) [6]	----	----	NR	SMR = 1.12 (0.96–1.31)
LeMaster et al. (2006) [7]	----	----	8	sRR = 1.14 (0.98–1.31)
Youakim S. (2006) [8]	4	sRR = 1.34 (0.82–2.06)	9	sRR = 1.08 (0.92–1.23)
Crawford et al. (2017) [10]	15	mRR = 1.04 (0.95–1.14)	----	----
Jalilian et al. (2019) [13]	15	SIRE = 0.97 (0.85–1.11)	9	SMRE = 1.06 (0.93–1.22)

**IARC:** International Agency for Research on Cancer; **mRR:** meta relative risk; **RE:** risk estimate; **SIRE:** summary incidence ratio estimate; **SMR:** standardized mortality ratio; **SMRE**: summary of mortality ratio estimate; **sRR:** summary relative risk. **Significant results are in bold.**

**Table 8:** Summary of risk estimates of urogenital cancers in firefighters from included SR.

Authors (Year) [Ref]	Cancer Incidence		Cancer Mortality	
	N Study	Risk Estimates (95% CI)	N Study	Risk Estimates (95% CI)
**Testicular cancer**				
Samet and Bhavsar (2005) [6]	----	----	NR	**SMR = 3.01 (1.02–8.90)**
LeMaster et al. (2006) [7]	4	**sRE = 2.02 (1.30–3.13)**	----	----
IARC (2010) [1]	6	**RE = 1.47 (1.20–1.80)**	----	----
Crawford et al. (2017) [10]	11	mRR = 1.22 (0.97–1.55)	----	----
Jalilian et al. (2019) [13]	9	**SIRE = 1.34 (1.08–1.68)**	----	----
**Prostate cancer**				
Samet and Bhavsar (2005) [6]	----	----	NR	**SMR = 1.27 (1.16–1.39)**
LeMaster et al. (2006) [7]	13	**sRE = 1.28 (1.15–1.43)**	----	----
IARC (2010) [1]	----	----	16	**RE = 1.30 (1.12–1.51)**
Crawford et al. (2010) [10]	19	**mRR = 1.13 (1.03–1.24)**	----	----
Sritharan et al. (2017) [11]	19	**mRE = 1.17 (1.08–1.28)**	10	mRE = 1.12 (0.92–1.36)
Jalilian et al. (2019) [13]	17	**SIRE = 1.15 (1.05–1.27)**	13	SMRE = 1.08 (0.92–1.27)
**Bladder cancer**				
Samet and Bhavsar (2005) [6]	----	----	NR	SMR = 1.23 (1.05–1.44)
LeMaster et al. (2006) [7]	----	----	11	sRE = 1.20 (0.97–1.48)
Youakim S. (2006) [8]	3	**sRR = 1.36 (1.01–1.80)**	10	sRR = 1.07 (0.95–1.15)
Crawford et al. (2017) [10]	16	**mRR = 1.12 (1.01–1.26)**	----	----
Jalilian et al. (2019) [13]	14	**SIRE = 1.12 (1.04–1.21)**	12	SMRE = 1.22 (0.93–1.60)
**Kidney cancer**				
Samet and Bhavsar (2005) [6]	----	----	NR	**SMR = 1.44 (1.23–1.70)**
LeMaster et al. (2006) [7]	----	----	12	sRE = 1.07 (0.78–1.46)
Youakim S. (2006) [8]	4	**sRR = 0.48 (0.19–0.98)**	9	**sRR = 1.22 (1.02–1.43)**
Crawford et al. (2017) [10]	16	**mRR = 1.16 (1.00–1.35)**	----	----
Jalilian et al. (2019) [13]	15	SIRE = 1.12 (0.93–1.36)	11	SMRE = 1.19 (0.90–1.58)

**IARC:** International Agency for Research on Cancer; **mRR:** meta relative risk; **RE:** risk estimate; **SIRE:** summary incidence ratio estimate; **SMR:** standardized mortality ratio; **SMRE**: summary of mortality ratio estimate; **sRE:** summary risk estimate; **sRR:** summary relative risk. **Significant results are in bold.**

**Table 9:** Summary of risk estimates of other types of cancer in firefighters from included SR.

Authors (Year) [Ref]	Cancer Incidence		Cancer Mortality	
	N Study	Risk Estimates (95%CI)	N Study	Risk Estimates (95%CI)
**Brain and nervous system cancer**				
Howe and Burch (1990) [3]	----	----	4	SMR = 1.43 (0.93–2.12)
Samet and Bhavsar (2005) [6]	----	----	NR	**SMR = 1.30 (1.10–1.51)**
Youakim S. (2006) [8]	6	sRR = 1.39 (0.90–2.05)	14	sRR = 1.09 (0.92–1.25)
Crawford et al. (2017) [10]	18	mRR = 1.17 (0.97–1.41)	----	----
Jalilian et al. (2019) [13]	13	SIRE = 1.07 (0.87–1.33)	14	SMRE = 1.25 (0.96–1.63)
**Male breast cancer**				
Jalilian et al. (2019) [13]	5	SIRE = 1.02 (0.47–2.25)	3	SMRE = 2.41 (0.65–9.48)
**Thyroid cancer**				
Jalilian et al. (2019) [13]	10	**SIRE = 1.22 (1.01–1.48)**	----	----
**Bones cancer**				
Jalilian et al. (2019) [13]	4	SIRE = 1.05 (0.52–2.13)	----	----
**Softt tissue sarcoma**				
Jalilian et al. (2019) [13]	4	SIRE = 1.11 (0.85–1.44)	----	----
**Eye cancer**				
Jalilian et al. (2019) [13]	2	SIRE = 1.12 (0.59–2.48)	----	----

**mRR:** meta relative risk; **SIRE:** summary incidence ratio estimate; **SMR:** standardized mortality ratio; **SMRE**: summary of mortality ratio estimate; **sRR:** summary relative risk. **Significant results are in bold.**

## Appendix A

**Table A1:** Included studies in selected systematic reviews.

Author	Year of Publication	Type of Study	Howe and Burch (1990) [3]	Samet (2005) [6]	Youakim (2006) [8]	LeMaster (2006) [7]	IARC (2010) [1]	Gomes (2011) [9]	Guidotti (2004) [2]	Sritharan (2017) [11]	Crawford (2017) [10]	IRSST (2018) [12]	Jalilian (2019) [13]
Mastromatteo, [23]	1959	C	X			X	X						X
Berg, [24]	1975	D				X	X						X
Milham, [25]	1976	D	X				X						
Williams, [26]	1977	D					X						
Musk, [27]	1978	C	X	X	X	X	X						X
Petersen, [28]	1980	D	X										
Lewis, [29]	1982	C	X										
Dubrow, [30]	1983	D				X	X						
Howe, [31]	1983	C	X										
Eliopulos, [32]	1984	C	X	X		X	X		X		X		X
Morton, [33]	1984	D			X	X	X						X
Blair, [34]	1985	D					X						X
Feuer, [35]	1986	D	X		X	X	X		X				X
Rosenstock, [36]	1987	C	X										
Vena, [15]	1987	C	X	X	X	X	X		X	X	X		X
Gallagher, [37]	1989	D	X				X						
Howe, [3]	1990	SR			X		X		X				
Heyer, [38]	1990	C	X	X	X		X		X				
Sama, [39]	1990	CC		X	X	X	X		X		X		X
Hansen, [40]	1990	D		X		X	X		X				X
Brownson, [41]	1990	CC					X						
Beaumont, [42]	1991	C		X	X	X	X		X	X	X		X
Grimes, [43]	1991	C		X	X	X	X			X	X		X
Demers, [44]	1992	C		X	X	X	X				X		X
Demers, [45]	1992	C			X	X	X		X	X	X		
Hrubec, [46]	1992	CC					X						
Demers [47]	1993	CC				X	X						
Giles, [48]	1993	C		X	X	X	X		X	X	X		
Guidotti, [49]	1993	C		X	X	X	X		X	X	X		X
Burnett, [50]	1994	CC		X	X	X			X		X		X
Aronson, [51]	1994	C		X	X	X	X		X	X	X		X
Tornling, [52]	1994	C		X	X	X	X			X	X		X
Demers, [53]	1994	C			X		X		X	X	X		X
Figgs, [54]	1995	CC			X	X							X
Deschamps, [55]	1995	C		X		X	X		X		X		X
Delahunt, [56]	1995	CC			X	X	X		X				X
Guidotti, [57]	1995	NR							X				
Muscat, [58]	1995	CC					X						
OPCS, [59]	1995	D					X						
Finkelstein, [60]	1995	CC					X						X
Bates, [61]	1995	C							X		X		
Firth, [62]	1996	C		X		X			X		X		X
Aronson, [63]	1996	CC							X				
Donnan, [64]	1996	C									X		
Ma, [65]	1998	D		X	X	X	X		X		X		X
Krstev, [66]	1998	CC					X		X	X			X
Carozza, [67]	2000	CC			X		X						
Baris, [68]	2001	C		X	X	X	X		X	X	X		X
Bates, [69]	2001	C			X	X	X		X	X	X		X
Goldberg, [70]	2001	CC			X								
Zeegers, [71]	2001	C							X				
Ma, [72]	2002	C							X				
Stang, [73]	2003	CC				X	X		X		X		X
Krishnan, [74]	2003	CC			X		X		X				X
Elci, [75]	2003	CC					X						X
Gaertner, [76]	2004	CC					X		X				X
Zhang, [77]	2004	C							X				
Zeegers, [78]	2004	C							X	X			X
Giordano, [79]	2004	C							X				
Ma, [80]	2005	C			X		X		X	X	X		X
Ma, [81]	2006	C					X		X	X	X		X
Bates, [82]	2006	CC							X				
LeMaster, [7]	2006	SR					X		X		X		
Youakim, [8]	2006	SR							X				
Bates, [83]	2007	CC					X	X	X		X	X	
Suarez, [84]	2007	CC							X				
Parent, [85]	2007	CC										X	
IARC, [86]	2007	SR									X		
Kang, [87]	2008	CC						X	X	X	X	X	X
t’Mannetje, [88]	2008	CC										X	
Glass, [89]	2009	C									X		
MacArthur, [90]	2009	CC										X	
Coggon, [91]	2009	C									X		
Graveling, [92]	2010	SR									X		
Heck, [93]	2010	CC										X	
Olsson, [94]	2010	CC										X	
Demers, [95]	2011	C							X				
Fang, [96]	2011	CC									X	X	
Zeig-Owens, [97]	2011	C									X		X
Corbin, [98]	2011	CC										X	X
Villeneuve, [99]	2011	CC										X	
Ahn, [100]	2012	C							X	X		X	X
Karami, [101]	2012	CC										X	X
Villeneuve, [102]	2012	CC										X	
Paget-Bailly, [103]	2013	CC									X	X	X
Santi, [104]	2013	CC										X	
Roelofs, [105]	2013	CC										X	
Daniels, [106]	2014	C							X	X	X	X	X
Pukala, [107]	2014	C								X		X	X
Ide, [108]	2014	C									X	X	
Amadeo, [109]	2015	C								X	X	X	X
Tsai, [110]	2015	CC								X	X	X	X
Ahn, [111]	2015	C									X	X	X
Daniels, [112]	2015	C									X	X	
Yip, [113]	2015	C									X		
Glass, [114]	2016	C								X		X	
Sritharan, [115]	2016	CC								X			
Sauvé, [116]	2016	CC								X			
Driscoll, [117]	2016	C									X		
Bigert, [118]	2016	CC										X	
Sritharan, [119]	2017	CC								X			X
Petersen, [120]	2018	C											X
Sritharan, [121]	2018	C								X			
Kullberg, [122]	2018	C											X

C: cohort; CC: case-control, D: descriptive; IARC: International Agency for Research on Cancer; IRSST: Institut de recherche Robert-Sauvé en santé et en sécurité du travail; NR: narrative review; OPCS: Office of Population Censuses and Surveys; SR: systematic review.

## Appendix B

**Table A2:** Preferred Reporting Items for Systematic Reviews and Meta-Analysis (PRISMA) statement.

Section/Topic	#	Checklist Item	Reported on Page #
**TITLE**			
Title	1	Identify the report as a systematic review, meta-analysis, or both.	1
**ABSTRACT**			
Structured summary	2	Provide a structured summary including, as applicable: background; objectives; data sources; study eligibility criteria, participants, and interventions; study appraisal and synthesis methods; results; limitations; conclusions and implications of key findings; systematic review registration number.	2
**INTRODUCTION**			
Rationale	3	Describe the rationale for the review in the context of what is already known.	3
Objectives	4	Provide an explicit statement of questions being addressed with reference to participants, interventions, comparisons, outcomes, and study design (PICOS).	3
**METHODS**			
Protocol and registration	5	Indicate if a review protocol exists, if and where it can be accessed (e.g., Web address), and, if available, provide registration information including registration number.	NR
Eligibility criteria	6	Specify study characteristics (e.g., PICOS, length of follow-up) and report characteristics (e.g., years considered, language, publication status) used as criteria for eligibility, giving rationale.	4
Information sources	7	Describe all information sources (e.g., databases with dates of coverage, contact with study authors to identify additional studies) in the search and date last searched.	3
Search	8	Present full electronic search strategy for at least one database, including any limits used, such that it could be repeated.	Appendix C
Study selection	9	State the process for selecting studies (i.e., screening, eligibility, included in systematic review, and, if applicable, included in the meta-analysis).	4
Data collection process	10	Describe method of data extraction from reports (e.g., piloted forms, independently, in duplicate) and any processes for obtaining and confirming data from investigators.	5
Data items	11	List and define all variables for which data were sought (e.g., PICOS, funding sources) and any assumptions and simplifications made.	5
Risk of bias in individual studies	12	Describe methods used for assessing risk of bias of individual studies (including specification of whether this was done at the study or outcome level), and how this information is to be used in any data synthesis.	5
Summary measures	13	State the principal summary measures (e.g., risk ratio, difference in means).	5
Synthesis of results	14	Describe the methods of handling data and combining results of studies, if done, including measures of consistency (e.g., I^2^) for each meta-analysis.	---
Risk of bias across studies	15	Specify any assessment of risk of bias that may affect the cumulative evidence (e.g., publication bias, selective reporting within studies).	5
Additional analyses	16	Describe methods of additional analyses (e.g., sensitivity or subgroup analyses, meta-regression), if done, indicating which were pre-specified.	--
**RESULTS**			
Study selection	17	Give numbers of studies screened, assessed for eligibility, and included in the review, with reasons for exclusions at each stage, ideally with a flow diagram.	6
Study characteristics	18	For each study, present characteristics for which data were extracted (e.g., study size, PICOS, follow-up period) and provide the citations.	Table 1 and Table 2
Risk of bias within studies	19	Present data on risk of bias of each study and, if available, any outcome level assessment (see item 12).	13 + Table 3
Results of individual studies	20	For all outcomes considered (benefits or harms), present, for each study: (a) simple summary data for each intervention group (b) effect estimates and confidence intervals, ideally with a forest plot.	Table 4, Table 5, Table 6, Table 7, Table 8 and Table 9
Synthesis of results	21	Present results of each meta-analysis done, including confidence intervals and measures of consistency.	----
Risk of bias across studies	22	Present results of any assessment of risk of bias across studies (see Item 15).	----
Additional analysis	23	Give results of additional analyses, if done (e.g., sensitivity or subgroup analyses, meta-regression [see Item 16]).	----
**DISCUSSION**			
Summary of evidence	24	Summarize the main findings including the strength of evidence for each main outcome; consider their relevance to key groups (e.g., healthcare providers, users, and policy makers).	24
Limitations	25	Discuss limitations at study and outcome level (e.g., risk of bias), and at review-level (e.g., incomplete retrieval of identified research, reporting bias).	24–26
Conclusions	26	Provide a general interpretation of the results in the context of other evidence, and implications for future research.	27
**FUNDING**			
Funding	27	Describe sources of funding for the systematic review and other support (e.g., supply of data); role of funders for the systematic review.	

## Appendix C

**Table A3:** Literature search strategies.

**MEDLINE (Pubmed)**	
**Search lines**	**Search strategies**
#1	(“Neoplasms”[Mesh] OR “Neoplasms”[tiab] OR “Neoplasms”[OT] OR “neoplasia”[tiab] OR “neoplasia”[OT] OR “neoplasias”[tiab] OR “neoplasias”[OT] OR “neoplasm”[tiab] OR “neoplasm”[OT] OR “tumor”[tiab] OR “tumor”[OT] OR “tumors”[tiab] OR “tumors”[OT] OR “tumour”[tiab] OR “tumour”[OT] OR “tumours”[tiab] OR “tumours”[OT] OR “cancer”[tiab] OR “cancer”[OT] OR “cancers”[tiab] OR “cancers”[OT] OR “malignancy”[tiab] OR “malignancy”[OT] OR “malignancies”[tiab] OR “malignancies”[OT])
#2	(“Firefighters”[Mesh] OR “firefighters”[tiab] OR “firefighters”[OT] OR “firefighter”[tiab] OR “firefighter”[OT] OR “Fire and Rescue Personnel”[tiab] OR “Fire and Rescue Personnel”[OT] OR “fire fighters”[tiab] OR “fire fighters”[OT] OR “fire fighter”[tiab] OR “fire fighter”[OT] OR “fire man”[tiab] OR “fire man”[OT] OR “fire men”[tiab] OR “fire men”[OT] OR “fireman”[tiab] OR “fireman”[OT] OR “firemen”[tiab] OR “firemen”[OT] OR “fire woman”[tiab] OR “fire woman”[OT] OR “fire women”[tiab] OR “fire women”[OT] OR “firewoman”[tiab] OR “firewoman”[OT] OR “firewomen”[tiab] OR “firewomen”[OT] OR “pompier”[tiab] OR “pompier”[OT] OR “pompiers”[tiab] OR “pompiers”[OT] OR “pompière”[tiab] OR “pompière”[OT] OR “pompières”[tiab] OR “pompières”[OT])
#3	(#1 AND #2)
#4	#3 AND (French[lang] OR English[lang])
**Embase**	
**Search lines**	**Search strategies**
#1	‘neoplasm’/exp OR ‘neoplasm’:ab,ti OR ‘neoplasia’:ab,ti OR ‘neoplasias’:ab,ti OR ‘neoplasms’:ab,ti OR ‘tumor’:ab,ti OR ‘tumors’:ab,ti OR ‘tumour’:ab,ti OR ‘tumours’:ab,ti OR ‘cancer’:ab,ti OR ‘cancers’:ab,ti OR ‘malignancy’:ab,ti OR ‘malignancies’:ab,ti
#2	‘fire fighter’/exp OR ‘fire fighter’:ti,ab OR ‘firefighter’:ti,ab OR ‘firefighters’:ti,ab OR ‘fire fighters’:ti,ab OR ‘fire and rescue personnel’:ti,ab OR ‘fire man’:ti,ab OR ‘fire men’:ti,ab OR ‘fireman’:ti,ab OR ‘firemen’:ti,ab OR ‘fire woman’:ti,ab OR ‘fire women’:ti,ab OR ‘firewoman’:ti,ab OR ‘firewomen’:ti,ab OR ‘pompier’:ti,ab OR ‘pompiers’:ti,ab OR ‘pompière’:ti,ab OR ‘pompières’:ti,ab
#3	(#1 AND #2)
#4	#3 AND ([article]/lim OR [article in press]/lim) AND ([english]/lim OR [french]/lim) AND [embase]/lim
**Cochrane Library**	
**Search lines**	**Search strategies**
#1	MeSH descriptor: [Neoplasms] explode all trees
#2	(“Neoplasms” OR “neoplasia” OR “neoplasias” OR “neoplasm” OR “tumor” OR “tumors” OR “tumour” OR “tumours” OR “cancer” OR “cancers” OR “malignancy” OR “malignancies”)
#3	(#1 OR #2)
#4	MeSH descriptor: [Firefighters] explode all trees
#5	(“firefighters” OR “firefighter” OR “Fire and Rescue Personnel” OR “fire fighters” OR “fire fighter” OR “fire man” OR “fire men” OR “fireman” OR “firemen” OR “fire woman” OR “fire women” OR “firewoman” OR “firewomen” OR “pompier” OR “pompiers” OR “pompière” OR “pompières”)
#6	(#4 OR #5)
#7	(#3 AND #6)
#8	#7 in Cochrane Reviews, Cochrane Protocols, Trials, Clinical Answers
**Centre for Reviews and Dissemination**	
**Search lines**	**Search strategies**
#1	MeSH descriptor: [Neoplasms] explode all trees
#2	(“Neoplasms” OR “neoplasia” OR “neoplasias” OR “neoplasm” OR “tumor” OR “tumors” OR “tumour” OR “tumours” OR “cancer” OR “cancers” OR “malignancy” OR “malignancies”)
#3	(#1 OR #2)
#4	MeSH descriptor: [Firefighters] explode all trees
#5	(“firefighters” OR “firefighter” OR “Fire and Rescue Personnel” OR “fire fighters” OR “fire fighter” OR “fire man” OR “fire men” OR “fireman” OR “firemen” OR “fire woman” OR “fire women” OR “firewoman” OR “firewomen” OR “pompier” OR “pompiers” OR “pompière” OR “pompières”)
#6	(#4 OR #5)
#7	(#3 AND #6)
**CINAHL Plus**	
**Search lines**	**Search strategies**
#1	(MH “Neoplasms”)
#2	TI ( (“Neoplasms” OR “neoplasia” OR “neoplasias” OR “neoplasm” OR “tumor” OR “tumors” OR “tumour” OR “tumours” OR “cancer” OR “cancers” OR “malignancy” OR “malignancies”) ) OR AB ( (“Neoplasms” OR “neoplasia” OR “neoplasias” OR “neoplasm” OR “tumor” OR “tumors” OR “tumour” OR “tumours” OR “cancer” OR “cancers” OR “malignancy” OR “malignancies”) )
#3	(#1 OR #2)
#4	(MH “Firefighters”)
#5	TI ( (“firefighters” OR “firefighter” OR “Fire and Rescue Personnel” OR “fire fighters” OR “fire fighter” OR “fire man” OR “fire men” OR “fireman” OR “firemen” OR “fire woman” OR “fire women” OR “firewoman” OR “firewomen” OR “pompier” OR “pompiers” OR “pompière” OR “pompières”) ) OR AB ( (“firefighters” OR “firefighter” OR “Fire and Rescue Personnel” OR “fire fighters” OR “fire fighter” OR “fire man” OR “fire men” OR “fireman” OR “firemen” OR “fire woman” OR “fire women” OR “firewoman” OR “firewomen OR “pompier” OR “pompiers” OR “pompière” OR “pompières”) )
#6	(#4 OR #5)
#7	(#3 AND #6)
**PsycNET**	
**Search lines**	**Search strategies**
#1	Index Terms: {Neoplasms} *Search Databases: PsycINFO, PsycARTICLES, PsycBOOKS, PsycTESTS*
#2	(Title: “Neoplasms” OR Title: “neoplasia” OR Title: “neoplasias” OR Title: “neoplasm” OR Title: “tumor” OR Title: “tumors” OR Title: “tumour” OR Title: “tumours” OR Title: “cancer” OR Title: “cancers” OR Title: “malignancy” OR Title: “malignancies”) OR (Abstract: “Neoplasms” OR Abstract: “neoplasia” OR Abstract: “neoplasias” OR Abstract: “neoplasm” OR Abstract: “tumor” OR Abstract: “tumors” OR Abstract: “tumour” OR Abstract: “tumours” OR Abstract: “cancer” OR Abstract: “cancers” OR Abstract: “malignancy” OR Abstract: “malignancies”) *Search Databases: PsycINFO, PsycARTICLES, PsycBOOKS, PsycTESTS*
#3	(#1 OR #2)
#4	(Title: “firefighters” OR Title: “firefighter” OR Title: “Fire and Rescue Personnel” OR Title: “fire fighters” OR Title: “fire fighter” OR Title: “fire man” OR Title: “fire men” OR Title: “fireman” OR Title: “firemen” OR Title: “fire woman” OR Title: “fire women” OR Title: “firewoman” OR Title: “firewomen” OR Title: “pompier” OR Title: “pompiers” OR Title: “pompière” OR Title: “pompières”) OR (Abstract: “firefighters” OR Abstract: “firefighter” OR Abstract: “Fire and Rescue Personnel” OR Abstract: “fire fighters” OR Abstract: “fire fighter” OR Abstract: “fire man” OR Abstract: “fire men” OR Abstract: “fireman” OR Abstract: “firemen” OR Abstract: “fire woman” OR Abstract: “fire women” OR Abstract: “firewoman” OR Abstract: “firewomen” OR Abstract: “pompier” OR Abstract: “pompiers” OR Abstract: “pompière” OR Abstract: “pompières”) *Search Databases: PsycINFO, PsycARTICLES, PsycBOOKS, PsycTESTS*
#5	(#3 AND #4)
**Web of Science**	
**Search lines**	**Search strategies**
#1	*(TI=(Neoplasms OR neoplasia OR neoplasias OR neoplasm OR tumor OR tumors OR tumour OR tumours OR cance OR cancers OR malignancy OR malignancies) OR TS=(Neoplasms OR neoplasia OR neoplasias OR neoplasm OR tumor OR tumors OR tumour OR tumours OR cancer OR cancers OR malignancy OR malignancies)) AND LANGUAGE: (English OR French) AND DOCUMENT TYPES: (Article OR Early Access OR Review)* *Indexes=SCI-EXPANDED, SSCI, A&HCI, CPCI-S, CPCI-SSH, ESCI Timespan=All years*
#2	*(TI=(firefighters OR firefighter OR Fire and Rescue Personnel” OR fire fighters OR fire fighter OR fire man OR fire men OR fireman OR firemen OR fire woman OR fire women OR firewoman OR firewomen OR pompier OR pompiers OR pompière OR pompières) OR TS=(firefighters OR firefighter OR Fire and Rescue Personnel” OR fire fighters OR fire fighter OR fire man OR fire men OR fireman OR firemen OR fire woman OR fire women OR firewoman OR firewomen OR pompier OR pompiers OR pompière OR pompières)) AND LANGUAGE: (English OR French) AND DOCUMENT TYPES: (Article OR Early Access OR Review)* *Indexes=SCI-EXPANDED, SSCI, A&HCI, CPCI-S, CPCI-SSH, ESCI Timespan=All years*
#3	(#1 AND #2)
**ABI/INFORM Global**	
**Search lines**	**Search strategies**
#1	MAINSUBJECT.EXACT(“Cancer”)
#2	ti((Neoplasms OR neoplasia OR neoplasias OR neoplasm OR tumor OR tumors OR tumour OR tumours OR cancer OR cancers OR malignancy OR malignancies)) OR ab((Neoplasms OR neoplasia OR neoplasias OR neoplasm OR tumor OR tumors OR tumour OR tumours OR cancer OR cancers OR malignancy OR malignancies))
#3	(#1 OR #2)
#4	MAINSUBJECT.EXACT(“Firefighters”)
#5	ti((firefighters OR firefighter OR Fire and Rescue Personnel OR fire fighters OR fire fighter OR fire man OR fire men OR fireman OR firemen OR fire woman OR fire women OR firewoman OR firewomen OR pompier OR pompiers OR pompière OR pompières)) OR ab((firefighters OR firefighter OR Fire and Rescue Personnel OR fire fighters OR fire fighter OR fire man OR fire men OR fireman OR firemen OR fire woman OR fire women OR firewoman OR firewomen OR pompier OR pompiers OR pompière OR pompières))
#6	(#4 OR #5)
#7	(#3 AND #6)
**Scopus**	
**Search lines**	**Search strategies**
#1	TITLE-ABS-KEY (“Neoplasms” OR “neoplasia” OR “neoplasias” OR “neoplasm” OR “tumor” OR “tumors” OR “tumour” OR “tumours” OR “cancer” OR “cancers” OR “malignancy” OR “malignancies”)
#2	TITLE-ABS-KEY(“firefighters” OR “firefighter” OR “Fire and Rescue Personnel” OR “fire fighters” OR “fire fighter” OR “fire man” OR “fire men” OR “fireman” OR “firemen” OR “fire woman” OR “fire women” OR “firewoman” OR “firewomen” OR “pompier” OR “pompiers” OR “pompière” OR “pompières”)
#3	#1 AND #2
#4	*#3 AND ( LIMIT-TO (DOCTYPE, “ar”) OR LIMIT-TO (DOCTYPE, “re”)*

**Table A4:** Grey literature search.

**Keywords** **English**: cancer, firefighter **French** cancer, pompier			
**Abbreviation**	**Name**	**Country**	**Websites**
**General websites**			
ACMTS	Agence canadienne des médicaments et des technologies de la santé	Canada	http://www.cadth.ca/fr
AHRQ	Agency for Healthcare Research and Quality	United States	http://www.ahrq.gov/
CDC	Centre for Disease Control and Prevention	United States	https://www.cdc.gov/
HAS	Haute Autorité de Santé	France	http://www.has-sante.fr/
HSAC	Health Services Assessment Collaboration	New Zealand	http://www.healthsac.net/aboutus/aboutus.htm
INESSS	Institut national d’excellence en santé et en services sociaux	Canada (Quebec)	http://www.inesss.qc.ca/
KCE	Centre fédéral d’expertise des soins de santé	Belgium	http://www.kce.fgov.be/
MSAC	Medical Services Advisory Committee	Australia	http://www.msac.gov.au/
NICE	National Institute for Health and Care Excellence	United Kingdom	http://www.nice.org.uk/
NIHR HTA	National Institute for Health Research Health Technology Assessment Programme	United Kingdom	https://www.nihr.ac.uk/explore-nihr/funding-programmes/health-technology-assessment.htm
OHTAC	Ontario Health Technology Advisory Committee	Canada (Ontario)	http://www.hqontario.ca/evidence
OMS	Organisation mondiale de la Santé	International	http://www.who.int/fr/
SIGN	Scottish Intercollegiate Guidelines Network	Scotland	http://www.sign.ac.uk/
VORTAL	HTAi vortal	United States	http://vortal.htai.org/?q=search_websites
**Specific websites and professional organizations**			
**Occupational health and safety**			
CNESST	Commission des normes, de l’équité, de la santé et de la sécurité du travail	Canada (Quebec)	https://www.cnesst.gouv.qc.ca/Pages/accueil.aspx
IRSST	Institut de recherche Robert-Sauvé en santé et en sécurité du travail	Canada (Quebec)	https://www.irsst.qc.ca/
	Réseau de santé publique au travail	Canada (Quebec)	http://www.santeautravail.qc.ca/
INSPQ	Institut national de santé publique du Québec	Canada (Quebec)	https://www.inspq.qc.ca/
CCOHS	Canadian Centre for Occupational Health and Safety	Canada	https://www.ccohs.ca
CREOD	The Centre for Research Expertise in Occupational Disease	Canada	http://creod.on.ca/
IWH	Institute for Work & Health	Canada	https://www.iwh.on.ca/
OSHA	Occupational Safety and Health Administration	United States	https://www.osha.gov/
INSHPO	International Network of Safety & Health Professional Organisations	International	http://www.inshpo.org/
SCSST	Société canadienne de la santé et de la sécurité au travail (SCSST)	Canada	https://www.csse.org/index.html
ASSP	American Society of Safety Professionals	United States	https://www.assp.org/
APHA	American Public Health Association	United States	https://www.apha.org/
ACOM	American College of Occupational and Environmental Medicine	United States	https://acoem.org/
ANZSOM	Australian and New Zealand Society of Occupational Medicine	Australia	https://www.anzsom.org.au/
BOHS	British Occupational Hygiene Society	United Kingdom	http://www.bohs.org/
ESF	European Safety Federation	Europe	https://eu-esf.org/
CARWH	Canadian Association for Research on Work and Health	Canada	http://www.carwh.ca/fr/default.html
AQHSST	Association québecoise pour l’hygiène, la santé et la sécurité du travail	Canada (Quebec)	https://www.aqhsst.qc.ca
HSE	Health and Safety Executive	United States	https://www.hse.gov.uk
	WorkSafe BC	Canada	https://www.worksafebc.com/fr
NIOSH	The National Institute for Occupational Safety and Health	United States	https://www.cdc.gov/niosh/index.htm
**Oncology**			
ASCO	American Society of Clinical Oncology	United States	https://www.asco.org/
BCCA	British Columbia Cancer Agency	Canada	http://www.bccancer.bc.ca/
CCO	Cancer Care Ontario	Canada	https://www.cancercareontario.ca/en
ESMO	European Society of Medical Oncology	Switzerland	https://www.esmo.org/
FNCLCC	Fédération nationale des Centres de lutte contre le cancer	France	http://www.unicancer.fr/
INC	Institut national du cancer	France	https://www.e-cancer.fr
NCI	National Cancer Institute	United States	https://www.cancer.gov/
NCCN	National Comprehensive Cancer Network	United States	https://www.nccn.org/
**Firefighting**			
IAFF	International Association of Fire Fighters	United States	https://iaff.org/
OPFFA	Ontario Professional Fire Fighters Association	Canada	http://www.opffa.org/
FDSOA	Fire Department Safety Officers Association	United States	https://www.fdsoa.org/
IAFC	International Association of Fire Chiefs	United States	https://www.iafc.org/
NVFC	National Volunteer Fire Council	United States	https://www.nvfc.org/
NWCG	National Wildfire Coordinating Group	United States	https://www.nwcg.gov/
CAFC	Canadian Association of Fire Chiefs	Canada	https://cafc.ca/
AFAC	Aboriginal Firefighters Association of Canada	Canada	https://www.ifmo.ca/afac-apac/
CF	Canadian Firefighter	Canada	https://www.cdnfirefighter.com/
VFABC	Volunteer Firefighters Association of BC	Canada	http://www.firebc.org/
OPFFA	Ottawa Professional Fire Fighters Association	Canada	http://www.ottawafirefighters.org/index.cfm?section=1
BCPFFA	British Columbia Professional Fire Fighters Association	Canada	https://www.bcpffa.net/
FSWO	Fire Service Women Ontario	Canada	https://www.fswo.ca/
CVFSA	Canadian Volunteer Fire Services Association	Canada	https://cvfsa.ca/
AFFA	Alberta Fire Fighters Association	Canada	http://www.albertafirefighters.com/
SPPQ	Syndicat des pompiers et pompières du Québec	Canada	http://www.spq-ftq.com/
ACSIQ	Association des chefs en sécurité incendie du Québec	Canada	https://www.acsiq.qc.ca/cms/
APEQ	Association des pompiers de l’est du Québec	Canada	http://info-apeq.ca/
APM	Association des pompiers de Montréal	Canada	https://www.adpm.qc.ca/
APSAM	Association paritaire pour la santé et la sécurité du travail, secteur Affaire municipale	Canada	https://www.apsam.com/
RAPQ	Regroupement des associations de pompiers du Québec	Canada	http://www.pompiersdequebec.org/
APPQ	Association des pompiers professionnels de Québec	Canada	http://www.pompiersdequebec.org/
IAWFES	International Association of Women in Fire and Emergency Services	United States	https://www.womeninfire.org/
**Other website**			
	Google Scholar (first 5 pages)	Canada	https://scholar.google.ca

## Appendix D

Excluded studies:

Do not have a systematic review methodology (n = 14)

- Institut de Recherche Robert-Sauvé en Santé et en Sécurité du Travail. *Risque de Leucémie chez les Pompiers. Rapport réalisé par D McGregor*; Rapport R-517; Institut de Recherche Robert-Sauvé en Santé et en Sécurité du Travail: Montréal, QC, Canada, 2007; 34p.
- Institut de Recherche Robert-Sauvé en Santé et en Sécurité du Travail. *Risques de Cancer du Côlon et du Rectum chez les Pompiers*; Rapport réalisé par D McGregor. Rapport R-515; Institut de Recherche Robert-Sauvé en Santé et en Sécurité du Travail: Montréal, QC, Canada, 2007; 38p.
- Institut de Recherche Robert-Sauvé en Santé et en Sécurité du Travail. Risque de Myélome Multiple et de Cancers des Voies Respiratoires, de L’œsophage, de L’estomac, du Pancréas, de la Prostate, des Testicules et de la peau chez les Pompiers; Rapport réalisé par D McGregor. Rapport R-521; Institut de Recherche Robert-Sauvé en Santé et en Sécurité du Travail: Montréal, QC, Canada, 2007; 52p.
- Institut de Recherche Robert-Sauvé en Santé et en Sécurité du Travail. *Risques de Tumeurs de la Vessie Urinaire chez les Pompiers*; Rapport réalisé par D McGregor. Rapport R-400; Institut de Recherche Robert-Sauvé en Santé et en Sécurité du Travail: Montréal, QC, Canada, 2005; 30p.
- Institut de Recherche Robert-Sauvé en Santé et en Sécurité du Travail. *Risques de Tumeurs du rein chez les Pompiers*; Rapport réalisé par D McGregor. Rapport R-398; Institut de Recherche Robert-Sauvé en Santé et en Sécurité du Travail: Montréal, QC, Canada, 2005; 30p.
- Institut de Recherche Robert-Sauvé en Santé et en Sécurité du Travail. *Risques de Tumeurs Cérébrales chez les Pompiers*; Rapport réalisé par D McGregor. Rapport R-396; Institut de Recherche Robert-Sauvé en Santé et en Sécurité du Travail: Montréal, QC, Canada, 2005; 29p.
- Beranger, R.; Le Cornet, C.; Schuz, J.; Fervers, B. Occupational and environmental exposures associated with testicular germ cell tumours: Systematic review of prenatal and life-long exposures. *PLOS One* 2013, 8, e77130.
- Firefighters Cancer Support Network. Taking Action against Cancer in the Fire Service. 2013. Available online: http://www.womeninfire.org/wp-content/uploads/2014/04/FCSN-report.pdf (accessed on 19 October 2019).
- Dow, M.; Kunz, K.; Garis, L.; Thomas, L. Firefighters and Cancer: Understanding Risk Factor in an Environment of Change. University of the Fraser Valley. 2015. Available online: https://www.ufv.ca/media/assets/criminal-justice-research/Firefighters-and-Cancer.pdf (accessed on 20 October 2019).
- Golden, A.L.; Markowitz, S.B.; Landrigan, P.J. The risk of cancer in firefighters. *Occup. Med.* **1995**, *10*, 803–820.
- Golka, K.; Weistenhofer, W. Fire fighters, combustion products, and urothelial cancer. *J. Toxicol. Environ. Health Part B Crit. Rev*. **2008**, *11*, 32–44.
- Guidotti, T.I. Evaluating causality for occupational cancers: The example of firefighters. *Occup. Med*. **2007**, *57*, 466–471.
- Scannell, C.H.; Balmes, J.R. Pulmonary effects of firefighting. *Occup. Med*. **1995**, *10*, 789–801.
- Sergentanis, T.N.; Zagouri, F.; Tsilimidos, G.; Tsagianni, A.; Tseliou, M.; Dimopoulos, M.A.; Psaltopoulou, T. Risk Factors for Multiple Myeloma: A Systematic Review of Meta-Analyses. *Clin. Lymphoma Myeloma Leuk.* **2015**, *15*, 563–577.

Review focusing on forest firefighters (n = 1)

• Domitrovich, J.W.; Broyles, G.A.; Ottmar, R.D.; Reinhardt, T.E.; Naeher, L.P.; Kleinman, M.T.; Christopher, E.M.; Olorunfemi, A. Wildland Fire Smoke Health Effects on Wildland Firefighters and the Public. 2017. Available online: https://www.firescience.gov/projects/13-1-02-14/project/13-1-02-14_final_report.pdf (accessed on 19 October 2019).

Does not present result on cancer (n = 1)

• Groot, E.; Caturay, A.; Khan, Y.; Copes, R. A systematic review of the health impacts of occupational exposure to wildland fires. *Int. J. Occup. Med. Environ. Health* **2019**, *32*, 121–140.

Commentary (n =3)

- Casjens, S.; Bruning, T.; Taeger, D. Meta-analysis of cancer risks of professional firefighters. *Int. J. Cancer* **2019**, *145*, 1701.
- Kawada, T. Cancer incidence and mortality among firefighters. *Int. J. Cancer* **2019**, *145*, 869.
- Fritschi, L.; Glass DC. Firefighters and cancer: Where are we and where to now? *Occup. Environ. Med.* **2014**, *71*, 525–526.

Original study (n = 1)

• Rushton, L.; Bagga, S.; Bevan, R.; Brown, T.; Cherrie, J.; Holmes, P.; Fortunato, L.; Hutchings, S.; Slack, R.; Van Tongeren, M.; et al.The burden of occupational cancer in Great Britain. Overview report prepared by the Health and Safety Laboratory, the Institute of Environment and Health, the Institute of Occupational Medicine and Imperial College London for the Health and Safety Executive. 2012. Available online: http://www.hse.gov.uk/research/rrpdf/rr931.pdf (accessed on 19 October 2019).

Thesis (n = 1)

• Genaidy, A.M. Cancer Risk among Firefighters: Epidemiological Evidence. Department of Environmental Health of the College of Medicine. Ph.D. Thesis, University of Cincinnati: Cincinnati, OH, USA, 2004.
